# Complex Liver Resections for Colorectal Metastases: Are They Safe in the Low-Volume, Resource-Poor Caribbean Setting?

**DOI:** 10.1155/2015/570968

**Published:** 2015-02-02

**Authors:** Shamir O. Cawich, Dexter A. W. Thomas, Chunilal Ramjit, Roderick Bhagan, Vijay Naraynsingh

**Affiliations:** Department of Clinical Surgical Sciences, University of the West Indies, St. Augustine Campus, St. Augustine, Trinidad and Tobago

## Abstract

*Introduction*. Although many authorities suggest that major liver resections should only be carried out in high-volume specialized centres, many patients in the Caribbean do not have access to these health care systems. *Presentation of a Case*. A 50-year-old woman with a solitary colorectal metastasis invading the inferior vena cava underwent an extended left hepatectomy with caval resection and reconstruction. Several technical maneuvers were utilized that were suited to the resource-poor environment. *Conclusion*. We suggest that good outcomes can still be attained in the resource-poor, low-volume centres once dedicated and appropriately trained teams are available.

## 1. Introduction

Over the past 2-3 decades, liver resections have become accepted as feasible and safe in dedicated high-volume centres with service centralization [1]. However, in the Caribbean several geographic [2], political [3], and logistic [4] factors limit service centralization. In addition, surgeons in the Caribbean setting have to adapt and improvise to deliver care in these resource-poor health care systems [2–5]. Caribbean surgeons, therefore, treat relatively small numbers of patients without service centralization in resource-poor health care systems.

Although some authorities have argued that major liver resections should not be carried out in these circumstances [1], most of these patients cannot afford care in developed countries with service centralization and well-supported health care systems. We argue that a high standard of care can still be delivered in these less than ideal circumstances once dedicated, trained health care teams perform these operations, even at low volumes.

## 2. Presentation of a Case

A 50-year-old woman with no comorbidities presented 2 years after a sigmoid colectomy for colorectal carcinoma. Histology revealed moderately differentiated adenocarcinoma with 5/12 nodes positive for metastatic disease. Circumferential and longitudinal margins were microscopically clear of disease. She completed adjuvant systemic therapy with Xeloda (capecitabine).

**Figure 1:**
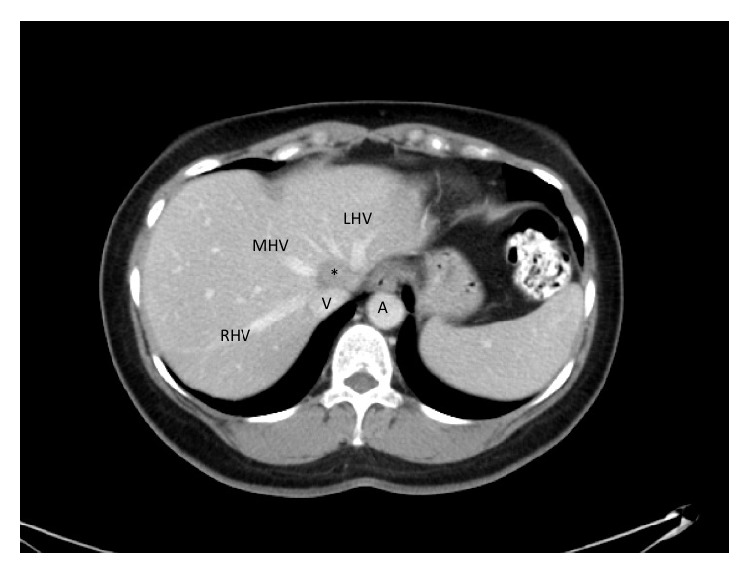
Axial CT images showing the IVC (V) receiving the LHV, MHV, and RHV. The solitary metastatic deposit (∗) is seen wedged between MHV and LHV and encroaches on the IVC. On axial cuts, the metastatic deposit is juxtaposed to, but does not appear to, involve the RHV.

**Figure 2:**
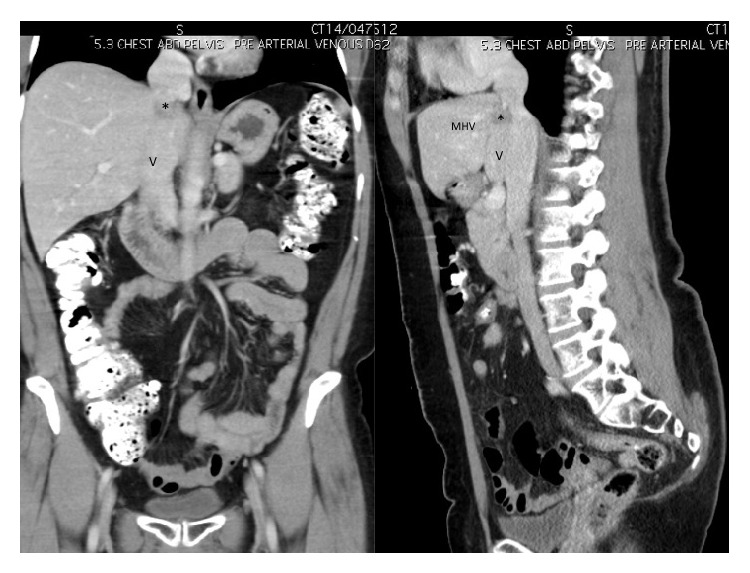
Coronal and sagittal reconstructed CT images showing the IVC (V) receiving the MHV. The solitary metastatic deposit (∗) is seen in segment IVa at the junction of the hepatic-caval confluence.

**Figure 3:**
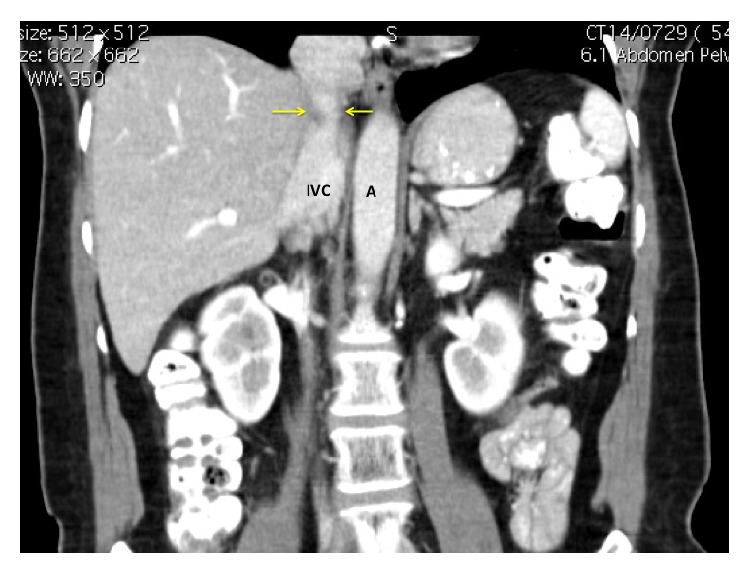
Coronal CT images marked with yellow arrows that point to the area of involvement of the retrohepatic IVC at the superior border of the liver. This area corresponds to the metastatic deposit on the axial images in Figure 1.

During surveillance, metastatic liver disease was detected. There was a single 2.5 cm metastatic deposit in segment IVa that involved left hepatic vein (LHV) and middle hepatic vein (MHV) and encroached on the inferior vena cava (IVC) anteriorly (Figures 1 and 2). Second line systemic therapy with IROX (irinotecan and oxaliplatin) was commenced but resulted in only minimal tumour regression. Although there was still a suspicion of IVC involvement after preoperative systemic therapy (Figure 3), she was taken to the operating theatre for exploration.

**Figure 4:**
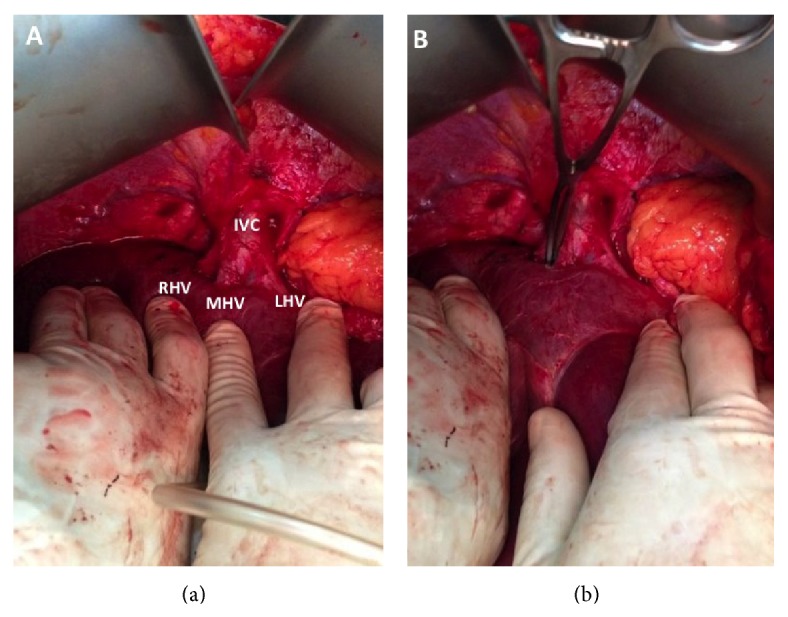
The hepatocaval junction is exposed in (a), allowing dissection of the space between MHV and RHV with a curved vascular clamp in (b).

The abdomen was accessed through an upper midline incision with transverse extension. No metastatic deposits were noted in the peritoneal cavity. The anterior layers of the coronary ligaments were incised to expose the hepatocaval junction (Figure 4). The metastatic deposit was not visible on inspection of the liver surface but intraoperative ultrasound confirmed the solitary deposit at segment IVa involving the MHV and LHV. Ultrasound also confirmed that the RHV and hepatocaval junction were uninvolved and the right hemiliver was free of metastases.

**Figure 5:**
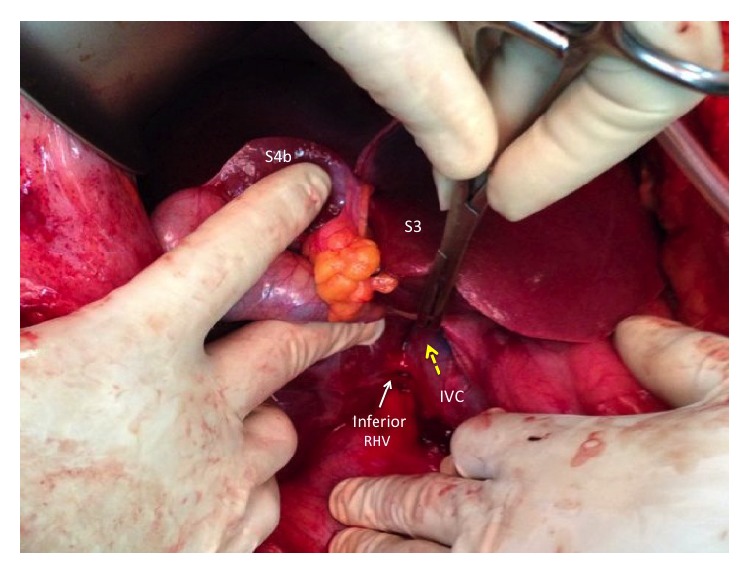
Segments III (S3) and IVb (S4b) of the liver are elevated to allow access to the IVC caudally. This allows access to the avascular plane on the IVC surface. A large vascular clamp bluntly dissects the avascular space in a cephalad direction as indicated by the broken yellow arrow. An inferior right hepatic vein has been identified and preserved.

**Figure 6:**
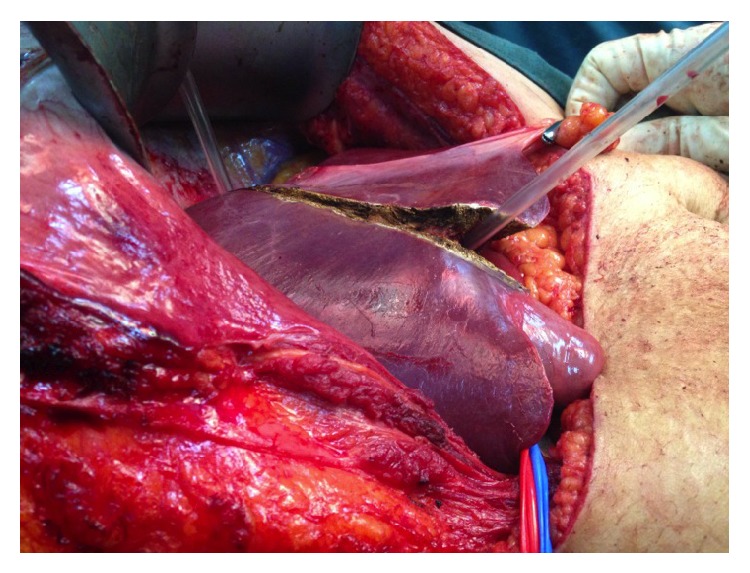
A nasogastric tube is used to execute the hanging maneuver to facilitate anterior parenchymal transection right down on to the IVC at the junction between RHV and MHV identified to be tumour-free on intraoperative ultrasound.

In preparation for a hanging maneuver, a curved vascular clamp was used for cephalad dissection between the MHV and RHV (Figure 4). Caudal dissection then followed over the avascular plane on the anterior IVC surface (Figure 5), preserving the inferior RHV. A large nasogastric tube was used to develop the plane and connect the planes from below. Traction on the nasogastric tube passed between RHV and MHV allowed us to perform the hanging manoeuvre (Figure 6).

**Figure 7:**
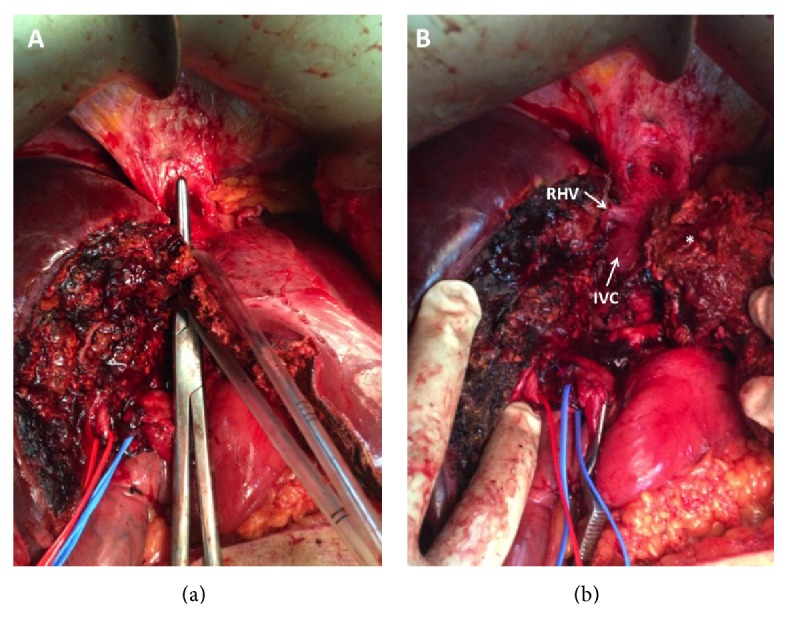
(a) The parenchyma was transected right down to the IVC and allowed the RHV to be preserved in order to maintain outflow from the future liver remnant.

**Figure 8:**
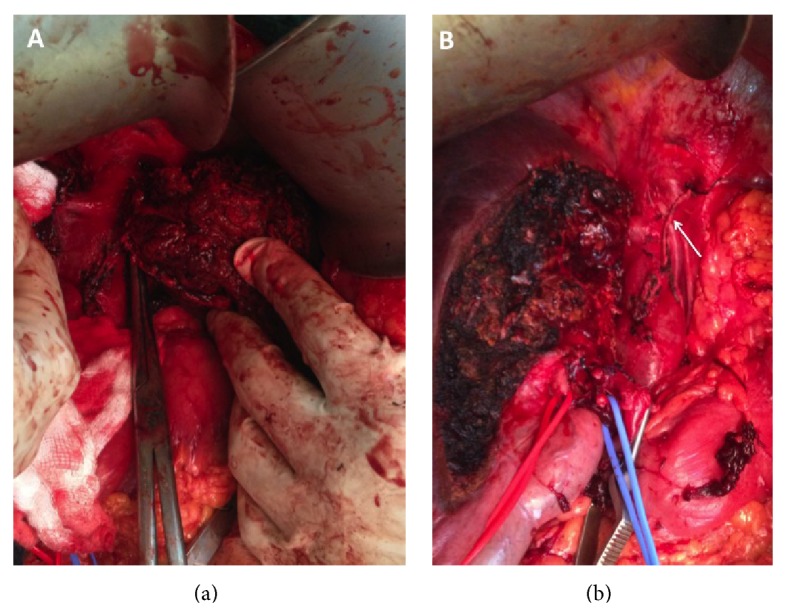
Anterior parenchymal transection allowed the metastasis to be approached from the right side, confirming IVC involvement. The left liver is now completely mobilized except at the point at which the metastatic deposit invades the cava. In (b) the left liver was completely excised and the IVC reconstructed (arrow).

The left coronary and triangular ligaments were then interrupted to expose the tumour posteriorly. A caudate lobectomy was performed because tumour extended into this lobe. The tumour also invaded the IVC at the hepatocaval junction on the left side. At this point, the hilar structures were controlled and divided in preparation for parenchymal transection. Anterior parenchymal transection was performed, skeletonizing and preserving the RHV at its entry into the IVC (Figure 7). This allowed the metastatic deposit to be approached from the right. In order to achieve a R0 resection, the IVC was temporarily clamped and a 2 cm segment of IVC was resected and reconstructed longitudinally (Figure 8). After reconstruction, there was narrowing of the IVC to about 70% of its normal diameter. Intraoperative blood loss was 650 mLs and no transfusion was required. The patient spent 36 hours in the intensive care unit and had an uneventful postoperative course. She was discharged from hospital at day 7 after the operation.

**Figure 9:**
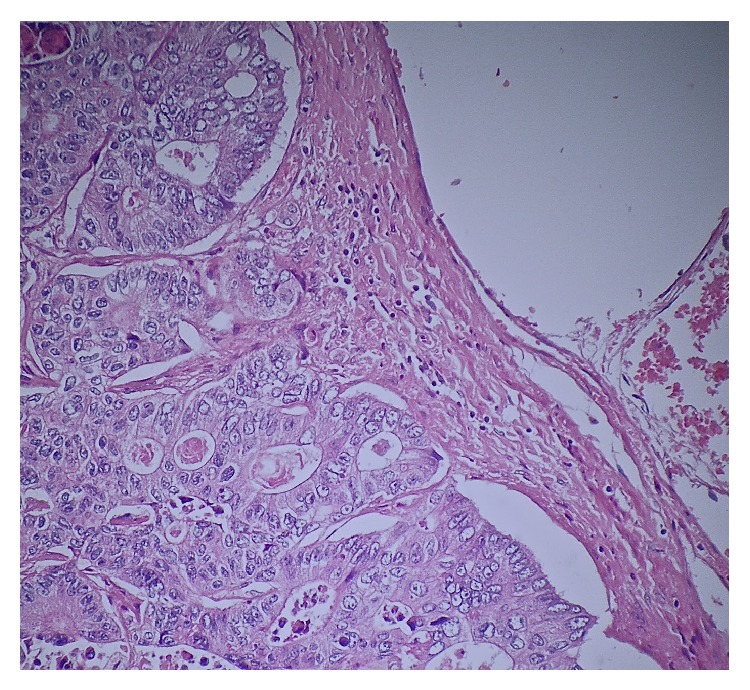
Microscopic examination of IVC at magnification ×10 with H&E stains. Moderately differentiated adenocarcinoma cells are noted to the left of the image, encroaching onto but not invading the endothelium of the IVC.

Pathologic examination of the specimen confirmed the presence of a 2.5 cm tumour deposit within the liver at the junction of LHV and MHV encroaching onto the IVC. On microscopy the moderately differentiated adenocarcinoma cells were noted to invade the IVC but had not yet penetrated the endothelium (Figure 9). All resection margins were clear with minimum margins of 0.5 mm at the IVC wall.

## 3. Discussion

It is now well accepted that major liver resections can be performed with acceptable morbidity and mortality in high-volume centers with dedicated hepatopancreatobiliary (HPB) teams and specialized supportive services [1]. In these settings, HPB surgeons have become quite aggressive in their pursuit of R0 resections, even in the face of tumour involving major structures such as the IVC.

Although major hepatectomy combined with IVC resection and reconstruction has now become feasible as a potentially curative resection for hepatic malignancy [6–10], most authorities suggest that these complex procedures should only be performed in high-volume centres [1]. In the Caribbean, however, this is not possible. Although there are approximately 6,500,000 persons living in the Anglophone Caribbean, they are scattered across many small island states each with small individual populations and few practitioners [4]. With three HPB surgical teams, Trinidad and Tobago has the largest HPB surgical service in the English speaking Caribbean. The HPB surgery service at Port of Spain General Hospital performs an average of 10 major liver resections each year, not qualifying as a high-volume service (>25 resections annually) according to international definitions [1]. Nevertheless, we have overcome this challenge by creating a dedicated specialist unit to treat HPB diseases with a multidisciplinary team approach [4]. The HPB team, staffed by one fellowship-trained HPB surgeon and two surgical house officers, has access to a general operating room with shared equipment and postoperative support from a multipurpose ICU and two gastroenterologists.

Although we performed IVC resection to achieve clear margins, histologic examination revealed that tumour did not penetrate the IVC endothelium. It is well recognized that IVC invasion is difficult to predict in preoperative imaging because this is a low-pressure vessel that can easily be compressed, especially by a large metastatic deposit [9, 10]. The metastatic deposit in this case was only 2.5 cm in diameter and so we maintained a suspicion that this may have been due to invasion rather than compression. However, the absence of histologic IVC invasion reinforces the fact that it is difficult to distinguish invasion and compression on preoperative imaging.

Maeba et al. [10] reported that 40% of patients with confirmed IVC invasion on preoperative imaging (including CT, MRI, and cavography) actually had histologic evidence of tumor invading the IVC. Despite using standard radiologic criteria (longitudinal compression > 50 mm, transverse compression extending > 50% of the circumference, lesions protruding into the IVC lumen, or the presence of well developed collaterals), it was difficult for them to accurately predict IVC invasion on imaging [10]. Transesophageal endoscopic ultrasonography may assist in distinguishing IVC invasion versus compression [11], but it was unavailable in our resource-poor setting.

In this case, we used the anterior parenchymal transection technique popularized by surgeons in Hong Kong [12–14]. Only by using this anterior transection technique were we able to approach the hepatocaval junction to achieve control from both sides of the tumour, thereby completing a safe controlled resection of the IVC with minimal blood loss. There are few disadvantages to anterior transection, including uncontrollable bleeding from vessels deep in the parenchyma and difficulty elevating the liver for effective manual compression. To circumvent these, we employed the hanging maneuver. The original hanging maneuver described by Belghiti et al. [15] in 2001 involved the passage of a tape in the avascular plane along the anterior surface of the IVC to suspend the liver. This serves to facilitate anterior parenchymal transection, bringing better control of bleeding from vessels deep within the parenhyma and a lower risk of tumour rupture [16]. In addition it guided the direction of anterior transection [16] that was indispensible in this case with metastatic deposits so close to the RHV.

To avoid the resultant haemodynamic consequences during IVC resection, many authorities advocate venovenous bypass [11, 17, 18]. An intraoperative decision was taken to proceed without bypass since selective clamping of the IVC as described by Togo et al. [11] allowed maintained flow through the native IVC. This maintained flow through both hepatic and systemic circulations [11]. If venovenous bypass was required, however, we were prepared to utilize the internal tube shunt method as described by Shimamura et al. [17]. Here an internal shunt tube is placed within the IVC lumen and banded above and below the liver. Shimamura et al. [17] utilized this method in seven major hepatectomies when caval resection was required with good outcomes. We were prepared to use the internal shunt tube as we thought it would be a good technique to use in the resource-poor settings such as ours.

Some authorities advocate IVC ligation after segmental resection, especially in cases of longstanding tumour obstruction when collaterals may be well developed [8, 19]. In these cases, they rely on collateral flow through the perivertebral plexus and the azygos vein [19]. However, there is the possibility of venous insufficiency in the lower limbs [20, 21] or acute renal failure [6, 21] if collateral flow is insufficient. Therefore, most authorities recommend IVC reconstruction after segmental resection [6, 8, 9]. Ohwada et al. [7] recommended IVC reconstruction with an interposed segment of PTFE graft when primary closure would result in >50% narrowing of the normal IVC diameter. In our case longitudinal reconstruction resulted in narrowing to approximately 70% of the normal IVC diameter. A smooth postoperative course was followed with no lower limb venous insufficiency nor renal compromise.

We acknowledge that the best results are obtained in high-volume centres with dedicated surgical teams and support services. However, many patients in developing countries do not have access to care in these high-volume centres. In these settings, a dedicated team approach with service centralization may still produce success in low-volume centres in developing Caribbean countries. It is important that the team consists of fellowship-trained HPB surgeons experienced in dealing with the complexities of liver resections. We believe that this, combined with a multidisciplinary team approach, is as important as the absolute number of resections performed on an annual basis in determining outcomes.

## 4. Conclusion

Although most authorities suggest that major liver resections should only be performed in high-volume centres, many patients in the Caribbean do not have access to these types of health care settings. We propose that good outcomes can also be attained in resource-poor centres once dedicated teams with appropriate support services and resources are made available, even at low volumes. The HPB surgeon in these settings must be familiar with techniques such as the hanging maneuver, internal shunt tube bypass, and selective caval clamping that may be well-suited for resource-poor environments.
